# 2-Meth­oxy-4-[1-(4-meth­oxy­phen­yl)-4,5-diphenyl-1*H*-imidazol-2-yl]phenol

**DOI:** 10.1107/S2414314626003160

**Published:** 2026-03-27

**Authors:** Seeralan Nagaraj, Nagarajan Loganathan

**Affiliations:** a School of Chemistry Bharathidasan University, Tiruchirappalli 620 024 Tamilnadu India; b UGC Faculty Recharge Programme, New Delhi, India; University of Aberdeen, United Kingdom

**Keywords:** crystal structure, Debus–Radziszewski reaction, criss-cross packing pattern

## Abstract

The title compound, C_29_H_24_N_2_O_3_, was synthesized *via* a four-component Debus–Radziszewski reaction, involving benzil, amine, aldehyde and ammonium acetate. The OH group of the vanillin substituent forms both an intra­molecular and an inter­molecular hydrogen bond, the latter generating [010] chains. The extended structure is consolidated by weak C—H⋯O and C—H⋯π inter­actions, generating a criss-cross motif.

## Abstract 2

In the title compound, which was synthesized *via* a four-component Debus–Radziszewski reaction, the OH group of the vanillin substituent forms both an intra­molecular and an inter­molecular hydrogen bond, the latter generating [010] chains. The extended structure is consolidated by weak C—H⋯O and C—H⋯π inter­actions, generating a criss-cross motif.

## Graphical abstract

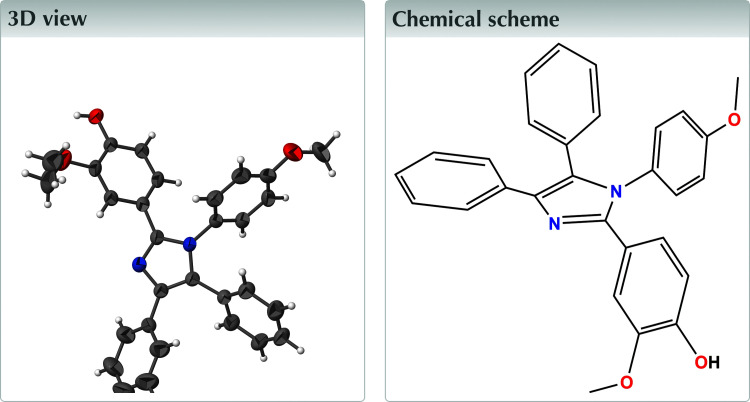

## Structure description

Imidazole is one of the widely studied classes of heterocyclic compounds owing to their immense importance in various physiological process and several enzymatic reactions (Ebel *et al.*, 2026 ▸). As part of our studies in this area, we reacted di­phenyl­ethane­dione (commonly known as benzil), 4-hy­droxy-3-meth­oxy­benzaldehyde (also known as vanillin), 4-meth­oxy­aniline and ammonium acetate (1:1:4:4 ratio) in glacial acetic acid under overnight reflux conditions. The isolated white solid was found to be the title compound,*C*_29_H_24_N_2_O_3_ (**1**) formed in 60–65% yield. This multicomponent reaction is also known as Debus–Radziszewski reaction and was originally reported between 1858–1882 (Debus, 1858 ▸; Radziszewski, 1882 ▸); while it was originally attempted to synthesize the 1,2,4-tris­ubstituted imidazole in the presence of ammonia, the use of ammonium acetate afforded the 1,2,4,5-tetra-substituted imidazole (Wang *et al.*, 2017 ▸).

**Figure 1:**
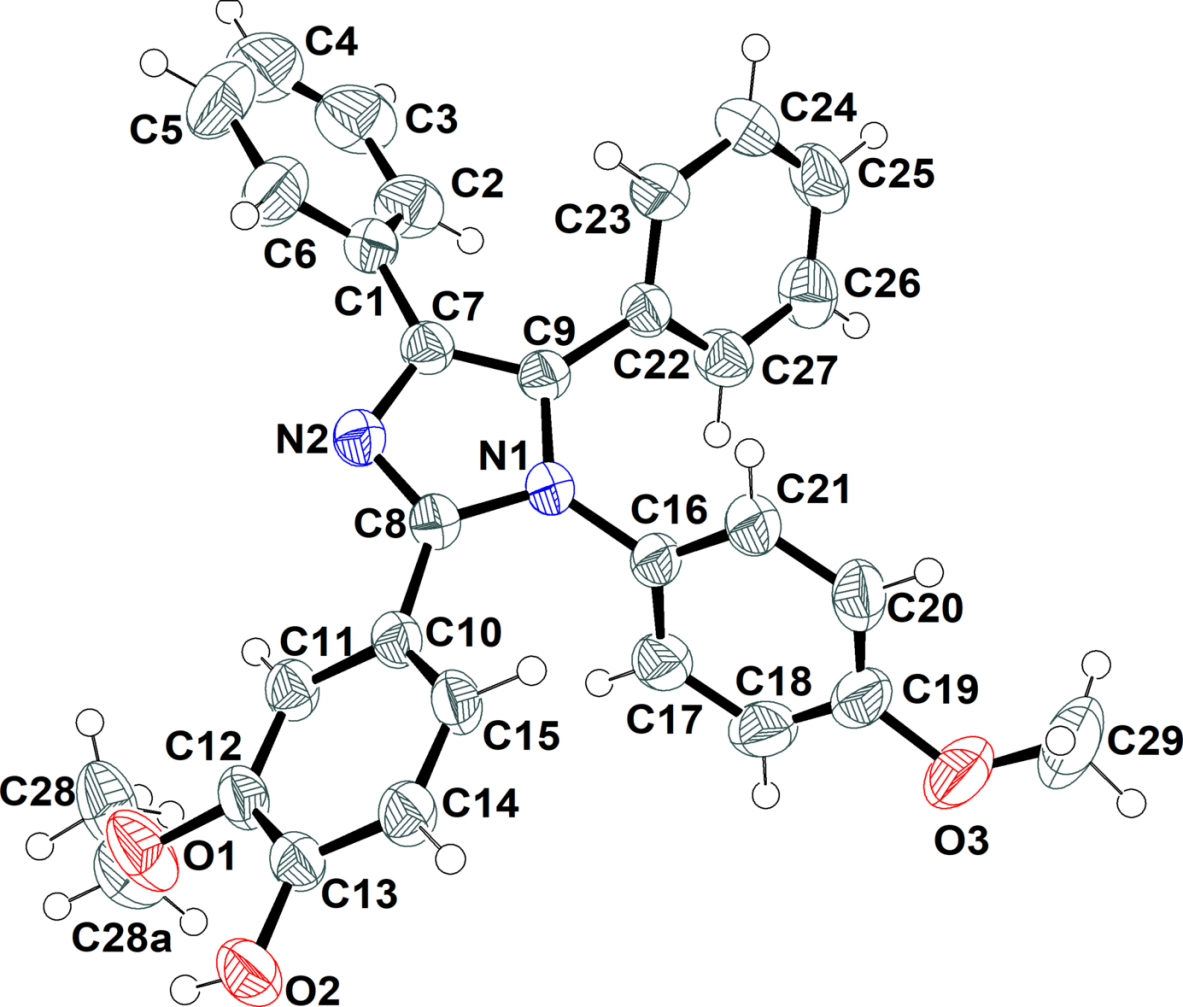
The mol­ecular structure of (**I**) showing 50% displacement ellipsoids.

Compound (**I**) crystallizes in the ortho­rhom­bic space group *P*2_1_2_1_2_1_ with one mol­ecule in the asymmetric unit. Earlier, we reported the analogous compound 1-(4-bromo­phen­yl)-4,5-di­phenyl-2-(1*H*-pyrrol-2-yl)-1*H*-imidazole (**II**) (Seeralan & Nagarajan, 2026 ▸) and the geometrical data for (**I**) are in good agreement with those for (**II**) as well as similar 1,2,4,5-tetra­substituted imidazole derivatives reported elsewhere (Xiao *et al.*, 2012 ▸; Zhao *et al.*, 2012 ▸). The mol­ecular structure of (**I**) (Fig. 1 ▸) shows that the central C7–C9/N1/N2 imidazole ring is substituted with C16–C21 4-meth­oxy­phenyl (anisole), C10–C15 4-hy­droxy-3-meth­oxy­phenyl (vanillin) and two phenyl groups (C1–C6 and C22–C27) at positions 1, 2, 4 and 5, respectively. The dihedral angles between the imidazole ring and the rings of the substituents are 62.66 (7), 40.37 (7), 42.22 (6) and 50.75 (8)°, respectively. The phenyl rings are not coplanar [56.55 (6)°] while the vanillin and 5-phenyl rings are almost perpendicular to each other [88.68 (7)°]; the anisole and 4-phenyl rings subtend a dihedral angle of 79.11 (8)°. An intra­molecular O2—H2*A*⋯O1 hydrogen bond (Table 1 ▸) closes an *S*(5) ring.

**Figure 2:**
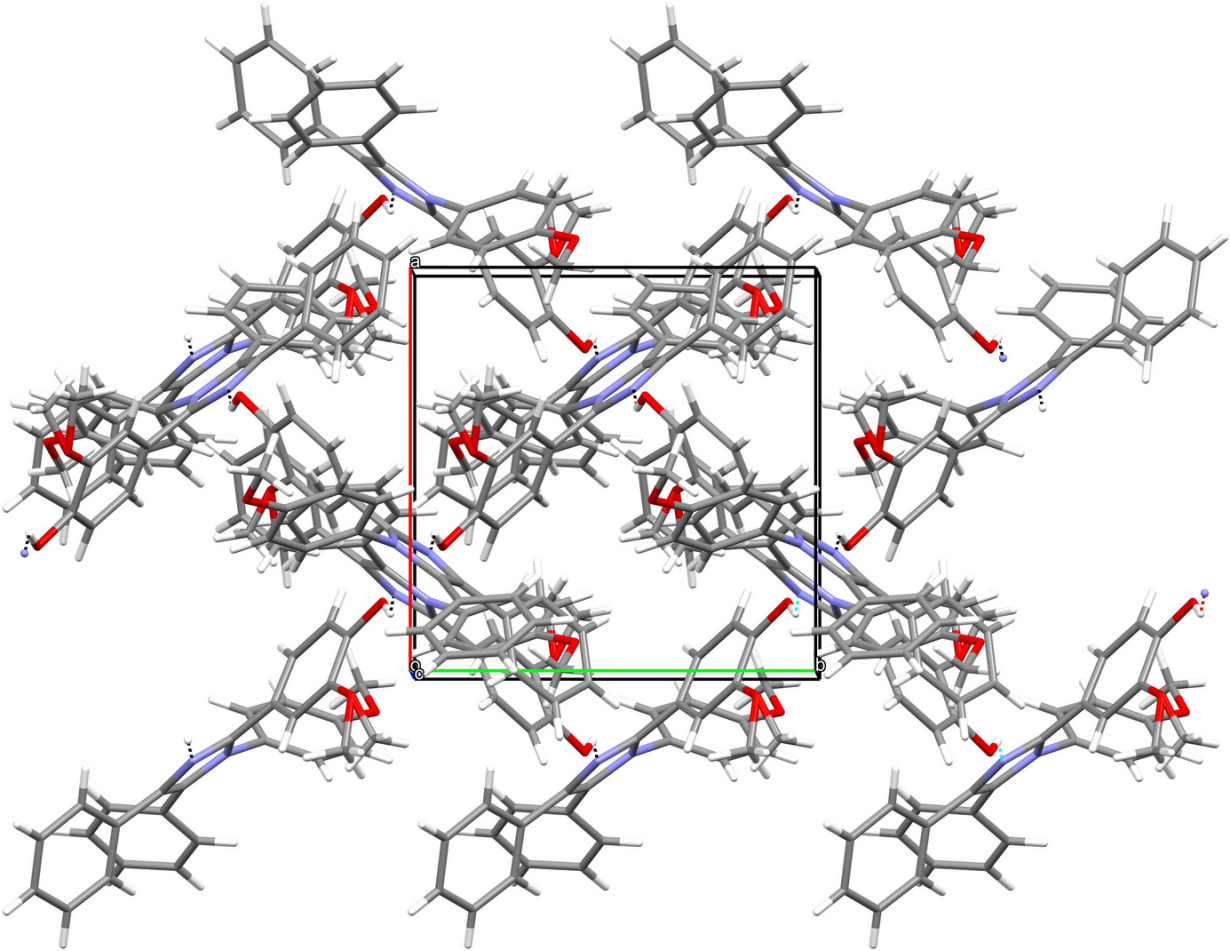
Perspective view along *c-*axis direction showing the supra­molecular architecture.

In the extended structure of (**I**), the same OH group also forms an inter­molecular link to N2, generating [010] chains and weak C—H⋯O and C—H⋯π links (Table 1 ▸) consolidate the structure, which resembles a criss-cross grid when viewed down [001] (Fig. 2 ▸).

## Synthesis and crystallization

The reaction of benzil (1.5324 g 7.28 mmol), vanillin (1.1076 g, 7.28 mmol), 4-meth­oxy­aniline (3.5470 g, 28.8 mmol) and ammonium acetate (3.7554 g, 28.8 mmol) in 35 ml of glacial acetic acid under overnight reflux condition, followed by quenching the reaction mixture in a crushed ice bath afforded a silvery precipitate that was filtered and purified by column chromatography using silica gel (hexane and eth­ylacetate (9:1) as eluent). Yield 65–70%, m.p. 225°C. FT–IR (cm^−1^) 3248(*br*), 2999(*w*), 2934(*w*), 2835(*w*), 1603(*s*), 1547(*s*), 1454(*s*), 1384(*s*), 1326(*m*), 1272(*w*), 1244(*m*), 1195(*s*), 1078(*s*), 1023(*s*), 974(*s*), 863(*s*), 787(*m*), 741(*s*) 620(*m*), 568(*m*). Yellow blocks of (**I**) were recrystallized from solution.

## Refinement

Crystal data, data collection and structure refinement details are summarized in Table 2 ▸. The C-bound hydrogen atoms were included in idealized positions (C—H = 0.93–0.96 Å) and the O—H proton was located in a difference-Fourier map. The C28 methyl group of the vanilin substituent is disordered over two sets of sites.

## Supplementary Material

Crystal structure: contains datablock(s) I. DOI: 10.1107/S2414314626003160/hb4560sup1.cif

Structure factors: contains datablock(s) I. DOI: 10.1107/S2414314626003160/hb4560Isup2.hkl

Supporting information file. DOI: 10.1107/S2414314626003160/hb4560Isup3.cml

CCDC reference: 2263383

Additional supporting information:  crystallographic information; 3D view; checkCIF report

## Figures and Tables

**Table 1:** Hydrogen-bond geometry (Å, °) *Cg*1 is the centroid of the C7–C9/N1/N2 ring.

*D*—H⋯*A*	*D*—H	H⋯*A*	*D*⋯*A*	*D*—H⋯*A*
O2—H2*A*⋯O1	0.82	2.28	2.718 (3)	114
O2—H2*A*⋯N2^i^	0.82	2.11	2.809 (3)	143
C4—H4⋯O2^ii^	0.93	2.55	3.379 (4)	148
C29—H29*C*⋯*Cg*1^iii^	0.96	2.98	3.526 (5)	118

Symmetry codes: (i) 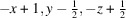; (ii) 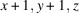; (iii) 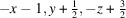.

**Table 2:** Experimental details

Crystal data	
Chemical formula	C_29_H_24_N_2_O_3_
*M*_r_	448.50
Crystal system, space group	Orthorhombic, *P*2_1_2_1_2_1_
Temperature (K)	300
*a*, *b*, *c* (Å)	9.9346 (4), 9.9936 (6), 23.9388 (14)
*V* (Å^3^)	2376.7 (2)
*Z*	4
Radiation type	Mo *K*α
μ (mm^−1^)	0.08
Crystal size (mm)	0.31 × 0.24 × 0.17

Data collection	
Diffractometer	Bruker D8 QUEST diffractometer with PHOTON II detector
Absorption correction	Multi-scan (*SADABS*; Krause *et al.*, 2015 ▸)
*T*_min_, *T*_max_	0.613, 0.746
No. of measured, independent and observed [*I* > 2σ(*I*)] reflections	30742, 5416, 4699
*R*_int_	0.036
(sin θ/λ)_max_ (Å^−1^)	0.650

Refinement	
*R*[*F*^2^ > 2σ(*F*^2^)], *wR*(*F*^2^), *S*	0.046, 0.102, 1.05
No. of reflections	5416
No. of parameters	317
H-atom treatment	H-atom parameters constrained
Δρ_max_, Δρ_min_ (e Å^−3^)	0.18, −0.16
Absolute structure	Flack *x* determined using 1682 quotients [(*I*^+^)−(*I*^−^)]/[(*I*^+^)+(*I*^−^)] (Parsons *et al.*, 2013 ▸)
Absolute structure parameter	0.3 (3)

Computer programs: *APEX4* and *SAINT* (Bruker, 2012 ▸), *SHELXT* (Sheldrick, 2015*a* ▸), *SHELXL2019/2* (Sheldrick, 2015*b* ▸), *ORTEP-3 for Windows*(Farrugia, 2012 ▸) and *publCIF* (Westrip, 2010 ▸).

## full crystallographic data

### (I) Crystal data

**Table:**

C_29_H_24_N_2_O_3_	*D*_x_ = 1.253 Mg m^−^^3^
*M**_r_* = 448.50	Mo *K*α radiation, λ = 0.71073 Å
Orthorhombic, *P*2_1_2_1_2_1_	Cell parameters from 9869 reflections
*a* = 9.9346 (4) Å	θ = 2.2–27.0°
*b* = 9.9936 (6) Å	µ = 0.08 mm^−^^1^
*c* = 23.9388 (14) Å	*T* = 300 K
*V* = 2376.7 (2) Å^3^	Block, yellow
*Z* = 4	0.31 × 0.24 × 0.17 mm
*F*(000) = 944	

### (I) Data collection

**Table:**

Bruker D8 QUEST diffractometer with PHOTON II detector	*R*_int_ = 0.036
Radiation source: i-mu-s microfocus source	θ_max_ = 27.5°, θ_min_ = 2.2°
φ and ω scans	*h* = −12→12
Absorption correction: multi-scan (SADABS; Krause *et al.*, 2015)	*k* = −12→12
*T*_min_ = 0.613, *T*_max_ = 0.746	*l* = −31→23
30742 measured reflections	5 standard reflections every 18 reflections
5416 independent reflections	intensity decay: none
4699 reflections with *I* > 2σ(*I*)	

### (I) Refinement

**Table:**

Refinement on *F*^2^	Hydrogen site location: inferred from neighbouring sites
Least-squares matrix: full	H-atom parameters constrained
*R*[*F*^2^ > 2σ(*F*^2^)] = 0.046	*w* = 1/[σ^2^(*F*_o_^2^) + (0.0408*P*)^2^ + 0.4588*P*] where *P* = (*F*_o_^2^ + 2*F*_c_^2^)/3
*wR*(*F*^2^) = 0.102	(Δ/σ)_max_ < 0.001
*S* = 1.05	Δρ_max_ = 0.18 e Å^−^^3^
5416 reflections	Δρ_min_ = −0.16 e Å^−^^3^
317 parameters	Absolute structure: Flack *x* determined using 1682 quotients [(*I*^+^)-(*I*^-^)]/[(*I*^+^)+(*I*^-^)] (Parsons *et al.*, 2013)
0 restraints	Absolute structure parameter: 0.3 (3)
Primary atom site location: structure-invariant direct methods	

### (I) Special details

**Table:**

Geometry. All esds (except the esd in the dihedral angle between two l.s. planes) are estimated using the full covariance matrix. The cell esds are taken into account individually in the estimation of esds in distances, angles and torsion angles; correlations between esds in cell parameters are only used when they are defined by crystal symmetry. An approximate (isotropic) treatment of cell esds is used for estimating esds involving l.s. planes.

### (I) Fractional atomic coordinates and isotropic or equivalent isotropic displacement parameters (Å^2^)

**Table:**

	*x*	*y*	*z*	*U*_iso_*/*U*_eq_	Occ. (<1)
O1	0.5463 (2)	0.1525 (3)	0.22051 (7)	0.0765 (7)	
O2	0.31259 (19)	0.0770 (2)	0.27049 (7)	0.0622 (5)	
H2A	0.342641	0.057331	0.239659	0.093*	
N1	0.68501 (19)	0.46222 (18)	0.43096 (7)	0.0394 (4)	
N2	0.70617 (19)	0.54653 (18)	0.34589 (8)	0.0407 (4)	
C1	0.8642 (3)	0.7350 (2)	0.36209 (10)	0.0461 (6)	
C2	0.9950 (3)	0.7539 (3)	0.38112 (12)	0.0618 (7)	
H2	1.029536	0.697210	0.408376	0.074*	
C3	1.0743 (4)	0.8552 (4)	0.36031 (16)	0.0822 (10)	
H3	1.161184	0.866786	0.373913	0.099*	
C4	1.0269 (5)	0.9378 (4)	0.32036 (16)	0.0920 (12)	
H4	1.081394	1.005382	0.306137	0.110*	
C5	0.8989 (5)	0.9220 (4)	0.30086 (16)	0.0981 (13)	
H5	0.866330	0.979097	0.273332	0.118*	
C6	0.8161 (4)	0.8204 (3)	0.32197 (12)	0.0731 (9)	
H6	0.728567	0.810924	0.308770	0.088*	
C7	0.7808 (2)	0.6230 (2)	0.38271 (9)	0.0392 (5)	
C8	0.6503 (2)	0.4513 (2)	0.37591 (9)	0.0387 (5)	
C9	0.7683 (2)	0.5741 (2)	0.43559 (9)	0.0386 (5)	
C10	0.5643 (2)	0.3459 (2)	0.35161 (9)	0.0386 (5)	
C11	0.5991 (2)	0.2961 (2)	0.29921 (9)	0.0440 (5)	
H11	0.678318	0.324721	0.282301	0.053*	
C12	0.5180 (2)	0.2053 (3)	0.27206 (9)	0.0460 (5)	
C13	0.3980 (2)	0.1630 (2)	0.29622 (9)	0.0437 (5)	
C14	0.3660 (2)	0.2086 (3)	0.34894 (10)	0.0464 (6)	
H14	0.288074	0.177873	0.366230	0.056*	
C15	0.4473 (2)	0.2990 (2)	0.37655 (9)	0.0443 (5)	
H15	0.423533	0.328613	0.412033	0.053*	
C16	0.6526 (2)	0.3704 (2)	0.47513 (9)	0.0393 (5)	
C17	0.6976 (3)	0.2397 (2)	0.47171 (10)	0.0504 (6)	
H17	0.746010	0.211452	0.440637	0.061*	
C18	0.6704 (3)	0.1517 (3)	0.51445 (12)	0.0618 (7)	
H18	0.700261	0.063711	0.512093	0.074*	
C19	0.5997 (3)	0.1931 (3)	0.56047 (11)	0.0614 (8)	
C20	0.5545 (3)	0.3230 (3)	0.56423 (10)	0.0564 (7)	
H20	0.506366	0.350888	0.595458	0.068*	
C21	0.5812 (2)	0.4127 (3)	0.52098 (9)	0.0470 (5)	
H21	0.550776	0.500543	0.523172	0.056*	
C22	0.8280 (2)	0.6210 (2)	0.48858 (9)	0.0409 (5)	
C23	0.8182 (3)	0.7555 (3)	0.50256 (11)	0.0554 (6)	
H23	0.767663	0.812753	0.480217	0.067*	
C24	0.8824 (3)	0.8051 (3)	0.54922 (11)	0.0654 (8)	
H24	0.875209	0.895430	0.558020	0.078*	
C25	0.9568 (3)	0.7223 (3)	0.58266 (11)	0.0638 (8)	
H25	1.001936	0.756490	0.613547	0.077*	
C26	0.9643 (3)	0.5877 (3)	0.57027 (11)	0.0592 (7)	
H26	1.012596	0.530508	0.593452	0.071*	
C27	0.9002 (2)	0.5375 (3)	0.52356 (10)	0.0489 (6)	
H27	0.905754	0.446652	0.515529	0.059*	
O3	0.5793 (3)	0.0972 (2)	0.60051 (10)	0.0965 (9)	
C28	0.6814 (8)	0.170 (2)	0.2004 (3)	0.072 (3)	0.58 (3)
H28A	0.689965	0.129657	0.164219	0.108*	0.58 (3)
H28B	0.701082	0.264003	0.197712	0.108*	0.58 (3)
H28C	0.743336	0.128719	0.225846	0.108*	0.58 (3)
C28A	0.6531 (17)	0.097 (2)	0.2082 (6)	0.077 (4)	0.42 (3)
H28D	0.649827	0.068399	0.169996	0.115*	0.42 (3)
H28E	0.726058	0.159089	0.213058	0.115*	0.42 (3)
H28F	0.666519	0.021125	0.232053	0.115*	0.42 (3)
C29	0.5122 (5)	0.1347 (5)	0.65042 (14)	0.1075 (15)	
H29A	0.504804	0.058414	0.674569	0.161*	
H29B	0.562577	0.203758	0.668903	0.161*	
H29C	0.423906	0.167462	0.641570	0.161*	

### (I) Atomic displacement parameters (Å^2^)

**Table:**

	*U*^11^	*U*^22^	*U*^33^	*U*^12^	*U*^13^	*U*^23^
O1	0.0651 (12)	0.1163 (17)	0.0480 (11)	−0.0288 (13)	0.0180 (9)	−0.0358 (12)
O2	0.0643 (11)	0.0801 (12)	0.0422 (9)	−0.0337 (10)	0.0078 (8)	−0.0151 (9)
N1	0.0474 (10)	0.0396 (10)	0.0312 (9)	−0.0035 (8)	−0.0033 (8)	−0.0003 (8)
N2	0.0426 (10)	0.0429 (10)	0.0365 (9)	−0.0005 (9)	−0.0034 (8)	0.0012 (8)
C1	0.0550 (14)	0.0435 (13)	0.0398 (12)	−0.0069 (11)	0.0031 (10)	−0.0009 (10)
C2	0.0537 (15)	0.0620 (16)	0.0697 (18)	−0.0084 (13)	0.0029 (14)	−0.0045 (15)
C3	0.070 (2)	0.082 (2)	0.095 (3)	−0.0299 (18)	0.0199 (19)	−0.017 (2)
C4	0.111 (3)	0.080 (2)	0.085 (2)	−0.048 (2)	0.029 (2)	−0.002 (2)
C5	0.140 (4)	0.080 (2)	0.075 (2)	−0.029 (2)	−0.002 (2)	0.032 (2)
C6	0.088 (2)	0.0657 (18)	0.0657 (18)	−0.0185 (18)	−0.0106 (17)	0.0181 (15)
C7	0.0403 (11)	0.0387 (11)	0.0388 (11)	0.0004 (9)	−0.0030 (9)	−0.0009 (9)
C8	0.0413 (11)	0.0410 (12)	0.0340 (11)	0.0000 (10)	−0.0020 (9)	−0.0002 (9)
C9	0.0431 (11)	0.0348 (11)	0.0377 (11)	0.0009 (9)	−0.0031 (9)	−0.0001 (9)
C10	0.0402 (11)	0.0424 (12)	0.0330 (11)	−0.0017 (10)	−0.0045 (9)	0.0008 (9)
C11	0.0400 (11)	0.0558 (14)	0.0362 (11)	−0.0088 (11)	0.0038 (9)	−0.0002 (10)
C12	0.0506 (13)	0.0578 (14)	0.0297 (10)	−0.0060 (11)	0.0034 (10)	−0.0054 (10)
C13	0.0459 (12)	0.0493 (13)	0.0359 (11)	−0.0092 (11)	−0.0033 (10)	−0.0023 (10)
C14	0.0429 (12)	0.0574 (14)	0.0389 (12)	−0.0101 (11)	0.0075 (10)	−0.0011 (11)
C15	0.0494 (13)	0.0542 (13)	0.0293 (11)	−0.0030 (11)	0.0027 (10)	−0.0047 (10)
C16	0.0426 (11)	0.0412 (12)	0.0340 (11)	−0.0066 (9)	−0.0073 (9)	0.0038 (9)
C17	0.0552 (14)	0.0475 (13)	0.0486 (13)	−0.0032 (12)	−0.0087 (12)	−0.0012 (11)
C18	0.0795 (19)	0.0424 (13)	0.0636 (18)	−0.0080 (13)	−0.0222 (15)	0.0080 (12)
C19	0.0768 (18)	0.0589 (16)	0.0485 (15)	−0.0289 (15)	−0.0228 (14)	0.0163 (13)
C20	0.0570 (15)	0.0769 (19)	0.0353 (12)	−0.0179 (14)	−0.0040 (11)	0.0026 (12)
C21	0.0517 (13)	0.0507 (13)	0.0384 (12)	−0.0037 (11)	−0.0059 (11)	0.0001 (11)
C22	0.0442 (12)	0.0440 (12)	0.0346 (11)	−0.0060 (10)	−0.0015 (9)	−0.0017 (9)
C23	0.0743 (17)	0.0452 (12)	0.0469 (13)	−0.0039 (13)	−0.0074 (13)	−0.0018 (11)
C24	0.091 (2)	0.0526 (15)	0.0528 (16)	−0.0132 (15)	−0.0064 (15)	−0.0130 (13)
C25	0.0635 (17)	0.082 (2)	0.0455 (14)	−0.0230 (15)	−0.0082 (13)	−0.0146 (14)
C26	0.0534 (15)	0.0758 (19)	0.0485 (15)	−0.0010 (14)	−0.0139 (12)	0.0020 (13)
C27	0.0495 (13)	0.0493 (13)	0.0478 (13)	0.0008 (11)	−0.0070 (11)	−0.0040 (11)
O3	0.142 (2)	0.0828 (15)	0.0649 (14)	−0.0480 (16)	−0.0225 (15)	0.0333 (12)
C28	0.061 (4)	0.113 (8)	0.043 (3)	0.003 (5)	0.009 (3)	−0.017 (4)
C28A	0.074 (7)	0.081 (9)	0.075 (6)	0.004 (7)	0.005 (5)	−0.014 (6)
C29	0.130 (3)	0.134 (3)	0.058 (2)	−0.064 (3)	−0.010 (2)	0.041 (2)

### (I) Geometric parameters (Å, º)

**Table:**

O1—C28A	1.232 (12)	C15—H15	0.9300
O1—C12	1.371 (3)	C16—C21	1.374 (3)
O1—C28	1.437 (10)	C16—C17	1.383 (3)
O2—C13	1.356 (3)	C17—C18	1.376 (4)
O2—H2A	0.8200	C17—H17	0.9300
N1—C8	1.367 (3)	C18—C19	1.371 (4)
N1—C9	1.395 (3)	C18—H18	0.9300
N1—C16	1.437 (3)	C19—O3	1.370 (3)
N2—C8	1.315 (3)	C19—C20	1.377 (4)
N2—C7	1.382 (3)	C20—C21	1.394 (3)
C1—C6	1.371 (4)	C20—H20	0.9300
C1—C2	1.390 (4)	C21—H21	0.9300
C1—C7	1.477 (3)	C22—C27	1.383 (3)
C2—C3	1.376 (4)	C22—C23	1.389 (4)
C2—H2	0.9300	C23—C24	1.379 (4)
C3—C4	1.348 (5)	C23—H23	0.9300
C3—H3	0.9300	C24—C25	1.368 (4)
C4—C5	1.364 (6)	C24—H24	0.9300
C4—H4	0.9300	C25—C26	1.380 (4)
C5—C6	1.401 (5)	C25—H25	0.9300
C5—H5	0.9300	C26—C27	1.380 (3)
C6—H6	0.9300	C26—H26	0.9300
C7—C9	1.363 (3)	C27—H27	0.9300
C8—C10	1.476 (3)	O3—C29	1.418 (5)
C9—C22	1.477 (3)	C28—H28A	0.9600
C10—C15	1.388 (3)	C28—H28B	0.9600
C10—C11	1.393 (3)	C28—H28C	0.9600
C11—C12	1.377 (3)	C28A—H28D	0.9600
C11—H11	0.9300	C28A—H28E	0.9600
C12—C13	1.391 (3)	C28A—H28F	0.9600
C13—C14	1.379 (3)	C29—H29A	0.9600
C14—C15	1.380 (3)	C29—H29B	0.9600
C14—H14	0.9300	C29—H29C	0.9600
			
C28A—O1—C12	124.3 (6)	C17—C16—N1	119.2 (2)
C12—O1—C28	116.5 (4)	C18—C17—C16	119.8 (3)
C13—O2—H2A	109.5	C18—C17—H17	120.1
C8—N1—C9	106.88 (18)	C16—C17—H17	120.1
C8—N1—C16	127.02 (18)	C19—C18—C17	120.4 (3)
C9—N1—C16	125.90 (17)	C19—C18—H18	119.8
C8—N2—C7	106.14 (17)	C17—C18—H18	119.8
C6—C1—C2	118.0 (3)	O3—C19—C18	115.3 (3)
C6—C1—C7	120.7 (2)	O3—C19—C20	124.4 (3)
C2—C1—C7	121.2 (2)	C18—C19—C20	120.3 (2)
C3—C2—C1	121.1 (3)	C19—C20—C21	119.7 (3)
C3—C2—H2	119.4	C19—C20—H20	120.2
C1—C2—H2	119.4	C21—C20—H20	120.2
C4—C3—C2	120.5 (3)	C16—C21—C20	119.6 (2)
C4—C3—H3	119.7	C16—C21—H21	120.2
C2—C3—H3	119.7	C20—C21—H21	120.2
C3—C4—C5	119.8 (3)	C27—C22—C23	118.3 (2)
C3—C4—H4	120.1	C27—C22—C9	122.5 (2)
C5—C4—H4	120.1	C23—C22—C9	119.1 (2)
C4—C5—C6	120.5 (3)	C24—C23—C22	120.7 (3)
C4—C5—H5	119.7	C24—C23—H23	119.7
C6—C5—H5	119.7	C22—C23—H23	119.7
C1—C6—C5	120.0 (3)	C25—C24—C23	120.5 (3)
C1—C6—H6	120.0	C25—C24—H24	119.8
C5—C6—H6	120.0	C23—C24—H24	119.8
C9—C7—N2	110.22 (19)	C24—C25—C26	119.5 (2)
C9—C7—C1	129.3 (2)	C24—C25—H25	120.2
N2—C7—C1	120.43 (19)	C26—C25—H25	120.2
N2—C8—N1	111.25 (19)	C25—C26—C27	120.2 (3)
N2—C8—C10	123.05 (19)	C25—C26—H26	119.9
N1—C8—C10	125.68 (19)	C27—C26—H26	119.9
C7—C9—N1	105.51 (18)	C26—C27—C22	120.7 (2)
C7—C9—C22	130.4 (2)	C26—C27—H27	119.6
N1—C9—C22	124.11 (19)	C22—C27—H27	119.6
C15—C10—C11	118.3 (2)	C19—O3—C29	118.3 (3)
C15—C10—C8	123.8 (2)	O1—C28—H28A	109.5
C11—C10—C8	117.76 (19)	O1—C28—H28B	109.5
C12—C11—C10	121.0 (2)	H28A—C28—H28B	109.5
C12—C11—H11	119.5	O1—C28—H28C	109.5
C10—C11—H11	119.5	H28A—C28—H28C	109.5
O1—C12—C11	124.0 (2)	H28B—C28—H28C	109.5
O1—C12—C13	115.7 (2)	O1—C28A—H28D	109.5
C11—C12—C13	120.4 (2)	O1—C28A—H28E	109.5
O2—C13—C14	118.8 (2)	H28D—C28A—H28E	109.5
O2—C13—C12	122.7 (2)	O1—C28A—H28F	109.5
C14—C13—C12	118.6 (2)	H28D—C28A—H28F	109.5
C13—C14—C15	121.3 (2)	H28E—C28A—H28F	109.5
C13—C14—H14	119.3	O3—C29—H29A	109.5
C15—C14—H14	119.3	O3—C29—H29B	109.5
C14—C15—C10	120.3 (2)	H29A—C29—H29B	109.5
C14—C15—H15	119.8	O3—C29—H29C	109.5
C10—C15—H15	119.8	H29A—C29—H29C	109.5
C21—C16—C17	120.3 (2)	H29B—C29—H29C	109.5
C21—C16—N1	120.5 (2)		
			
C6—C1—C2—C3	−0.1 (4)	C10—C11—C12—C13	1.1 (4)
C7—C1—C2—C3	177.5 (3)	O1—C12—C13—O2	−1.0 (4)
C1—C2—C3—C4	−0.7 (5)	C11—C12—C13—O2	177.9 (2)
C2—C3—C4—C5	0.8 (6)	O1—C12—C13—C14	177.7 (2)
C3—C4—C5—C6	−0.1 (6)	C11—C12—C13—C14	−3.4 (4)
C2—C1—C6—C5	0.8 (4)	O2—C13—C14—C15	−178.3 (2)
C7—C1—C6—C5	−176.8 (3)	C12—C13—C14—C15	3.0 (4)
C4—C5—C6—C1	−0.8 (6)	C13—C14—C15—C10	−0.2 (4)
C8—N2—C7—C9	−0.7 (2)	C11—C10—C15—C14	−2.1 (3)
C8—N2—C7—C1	176.2 (2)	C8—C10—C15—C14	174.3 (2)
C6—C1—C7—C9	−141.3 (3)	C8—N1—C16—C21	−121.5 (3)
C2—C1—C7—C9	41.1 (4)	C9—N1—C16—C21	64.4 (3)
C6—C1—C7—N2	42.4 (3)	C8—N1—C16—C17	60.4 (3)
C2—C1—C7—N2	−135.2 (2)	C9—N1—C16—C17	−113.7 (3)
C7—N2—C8—N1	0.1 (2)	C21—C16—C17—C18	0.1 (4)
C7—N2—C8—C10	−178.6 (2)	N1—C16—C17—C18	178.2 (2)
C9—N1—C8—N2	0.6 (3)	C16—C17—C18—C19	−0.3 (4)
C16—N1—C8—N2	−174.4 (2)	C17—C18—C19—O3	−179.6 (2)
C9—N1—C8—C10	179.2 (2)	C17—C18—C19—C20	0.4 (4)
C16—N1—C8—C10	4.2 (4)	O3—C19—C20—C21	179.9 (2)
N2—C7—C9—N1	1.1 (2)	C18—C19—C20—C21	−0.1 (4)
C1—C7—C9—N1	−175.6 (2)	C17—C16—C21—C20	0.2 (3)
N2—C7—C9—C22	−180.0 (2)	N1—C16—C21—C20	−177.9 (2)
C1—C7—C9—C22	3.4 (4)	C19—C20—C21—C16	−0.1 (4)
C8—N1—C9—C7	−1.0 (2)	C7—C9—C22—C27	−126.7 (3)
C16—N1—C9—C7	174.1 (2)	N1—C9—C22—C27	52.1 (3)
C8—N1—C9—C22	180.0 (2)	C7—C9—C22—C23	49.6 (4)
C16—N1—C9—C22	−4.9 (3)	N1—C9—C22—C23	−131.6 (2)
N2—C8—C10—C15	−139.4 (2)	C27—C22—C23—C24	2.0 (4)
N1—C8—C10—C15	42.1 (3)	C9—C22—C23—C24	−174.4 (2)
N2—C8—C10—C11	37.0 (3)	C22—C23—C24—C25	−0.2 (5)
N1—C8—C10—C11	−141.5 (2)	C23—C24—C25—C26	−1.7 (5)
C15—C10—C11—C12	1.6 (3)	C24—C25—C26—C27	1.8 (4)
C8—C10—C11—C12	−175.0 (2)	C25—C26—C27—C22	0.1 (4)
C28A—O1—C12—C11	53.2 (16)	C23—C22—C27—C26	−1.9 (4)
C28—O1—C12—C11	14.6 (9)	C9—C22—C27—C26	174.4 (2)
C28A—O1—C12—C13	−127.9 (16)	C18—C19—O3—C29	177.1 (3)
C28—O1—C12—C13	−166.5 (9)	C20—C19—O3—C29	−2.9 (4)
C10—C11—C12—O1	180.0 (2)		

### (I) Hydrogen-bond geometry (Å, º)

*Cg*1 is the centroid of the C7–C9/N1/N2 ring.

**Table:**

*D*—H···*A*	*D*—H	H···*A*	*D*···*A*	*D*—H···*A*
O2—H2*A*···O1	0.82	2.28	2.718 (3)	114
O2—H2*A*···N2^i^	0.82	2.11	2.809 (3)	143
C4—H4···O2^ii^	0.93	2.55	3.379 (4)	148
C29—H29*C*···*Cg*1^iii^	0.96	2.98	3.526 (5)	118

Symmetry codes: (i) −*x*+1, *y*−1/2, −*z*+1/2; (ii) *x*+1, *y*+1, *z*; (iii) −*x*−1, *y*+1/2, −*z*+3/2.
